# Primordial Follicle Transplantation within Designer Biomaterial Grafts Produce Live Births in a Mouse Infertility Model

**DOI:** 10.1038/srep17709

**Published:** 2015-12-03

**Authors:** E. Kniazeva, A. N. Hardy, S. A. Boukaidi, T. K. Woodruff, J. S. Jeruss, L. D. Shea

**Affiliations:** 1 Department of Chemical and Biological Engineering, McCormick School of Engineering, Northwestern University, Evanston, IL 60208, USA; 2 Department of Surgical Oncology, Fox Chase Cancer Center, Philadelphia, PA 19111, USA; 3 Department of Obstetrics and Gynecology and Reproductive Medicine, CHU de Nice, Archet 2 Hospital, Nice, France; 4 Department of Obstetrics and Gynecology, Institute for Women’s Health Research, Feinberg School of Medicine, Northwestern University, Chicago, IL 60611, USA; 5 Department of Surgery, University of Michigan, Ann Arbor, MI 48109, USA; 6 Department of Biomedical Engineering, University of Michigan, Ann Arbor, MI 48109, USA

## Abstract

The gonadotoxic effects of chemotherapy and radiation may result in premature ovarian failure in premenopausal oncology patients. Although autotransplantation of ovarian tissue has led to successful live births, reintroduction of latent malignant cells inducing relapse is a significant concern. In this report, we investigated the design of biomaterial grafts for transplantation of isolated ovarian follicles as a means to preserve fertility. Primordial and primary ovarian follicles from young female mice were extracted and encapsulated into biomaterials for subsequent transplantation into adult mice. Among the formulations tested, aggregated follicles encapsulated within fibrin had enhanced survival and integration with the host tissue following transplantation relative to the fibrin-alginate and fibrin-collagen composites. All mice transplanted with fibrin-encapsulated follicles resumed cycling, and live births were achieved only for follicles transplanted within VEGF-loaded fibrin beads. The extent to which these procedures reduce the presence of metastatic breast cancer cells among the isolated follicles was evaluated, with significantly reduced numbers of cancer cells present relative to intact ovaries. This ability to obtain live births by transplanting isolated primordial and primary follicles, while also reducing the risk of re-seeding disease relative to ovarian tissue transplantation, may ultimately provide a means to preserve fertility in premenopausal oncology patients.

Recent advances in cancer treatment have led to a marked improvement in survival rates. Simultaneously, cancer treatments are not without comorbidities, notably for women of reproductive age, who may survive cancer but undergo premature ovarian failure secondary to therapeutic exposure^1^,^2^. Patient concerns about future fertility ranked second only to questions about mortality, and greatly influenced decision-making regarding treatment^3^,^4^. Many chemotherapeutic regimens are fertility-threatening, in particular the alkylating agents and platinum-based drugs, which have been linked to infertility through DNA damage to the oocyte^5^,^6^,^7^,^8^,^9^,^10^. In addition, ionizing radiation to the pelvis, a therapeutic component of some pediatric colorectal, gynecologic, and hematologic cancers, is known to be gonadotoxic, with an effective sterilizing dose of less than 20 Gy^11^,^12^,^13^,^14^,^15^,^16^.

A number of options for fertility preservation have been developed; however, some techniques are not feasible secondary to the patient’s disease and condition, age, or relationship status^8^,^17^,^18^,^19^,^20^,^21^,^22^. A promising and emerging approach for the restoration of endocrine function and preservation of fertility, is the cryopreservation and autotransplantation of ovarian tissue^23^. This technique could be applied for patients losing ovarian function as a consequence of autoimmune or genetic disorders or chemotherapy treatment. While ovarian tissue transplantation has resulted in fertility preservation in primates^24^ and in humans (>60 live births to date)^25^,^26^,^27^,^28^,^29^,^30^,^31^,^32^,^33^, this technique can present a potential risk for reintroduction of malignant cells^34^, and has previously resulted in cancer relapse^35^. The transplantation of isolated ovarian follicles has the potential to restore ovarian function and fertility, yet also may limit the risk of re-seeding cancer cells^25^. While the aforementioned successes support the potential of the field, these approaches have not been widely implemented secondary to factors such as the relatively nascent stage of the clinical field, relatively small numbers of fertility clinics with the necessary expertise, and patient and disease specific factors. The development of robust technologies for efficiently maturing early stage follicles to produce fertilizable oocytes may contribute to overcoming some of these challenges.

Herein, we investigated the design of biomaterial grafts for the transplantation of primordial and primary follicles that can promote follicle engraftment, minimize follicle loss, and restore endocrine function. Additionally, these grafts were investigated for their support of follicle recruitment and maturation, facilitating the production of an oocyte capable of being fertilized and with the resulting embryo developing into a viable offspring. Three biomaterial combinations (fibrin, fibrin/alginate, and fibrin/collagen) were investigated for their effect on follicle survival and development. Such biomaterial systems have been used to encapsulate and culture secondary follicles *in vitro*, demonstrating that oocytes could be retrieved, fertilized, and implanted to produce live births in a mouse model^36^,^37^,^38^, as well as support development of nonhuman primate follicles^39^,^40^,^41^ and human secondary^39^ and pre-antral follicles^42^. Herein, these materials were employed for the encapsulation and transplantation of early stage follicles (primordial and primary, with some secondary), yet the material design was altered due to the significantly different requirements for *in vivo* transplantation relative to *in vitro* culture (e.g., integration with host tissue). Primordial and primary follicles were targeted as they comprise a large fraction of the ovarian follicle pool in fertile mice and have the great potential to survive cryopreservation^38^,^43^,^44^,^45^. Mice transplanted with primordial follicles encapsulated within biomaterials were investigated for their ability to resume cycling and to achieve a pregnancy. Furthermore, we evaluated the efficacy of our procedures in reducing the number of associated cancer cells relative to transplanted ovarian tissue using mice transplanted with human metastatic breast cancer cells^46^. Methods for follicle transplantation could have an important impact on the field of fertility preservation by extending transplantation-based options to cancer patients while minimizing the risk of subsequent ovarian tissue-transplant related cancer relapse.

## Results

### *In Vitro* Follicle Culture

**Figure 1:**
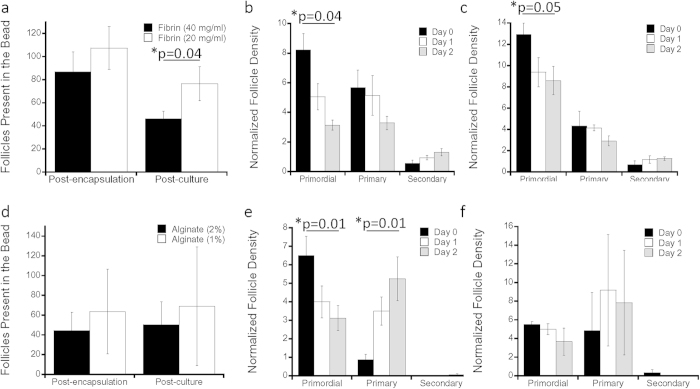
*In Vitro* Follicle Culture in Fibrin and Alginate. (**a**) Average absolute total number of follicles present in the bead are quantified for post-encapsulation and post-culture in 40 mg/ml and 20 mg/ml fibrin and 2% and 1% alginate (**d**), where error bars represent propagated error, with p-value determined by two-tailed Student’s t-test and n is as indicated in (**b**,**c**,**e**,**f**); follicle quantification in terms of their maturation stage (primordial, primary or secondary) vs. average normalized follicle density with (**b**) 40 mg/ml fibrin (n = 4); (**c**) 20 mg/ml fibrin (n = 4); (**e**) 2% alginate (n = 8); (**f**) 1% alginate (n = 4) over days 0, 1, 2, where error bars represent s.e.m. with p-value determined by two-tailed Student’s t-test; * indicate p ≤ 0.05, for (**b**,**c**) and (**e**) comparison is between day 0 and day 2.

The initial study investigated multiple material compositions for encapsulation of primordial and primary follicles into hydrogel beads, since the surrounding environment characteristics are important factors that affect proper follicle development^47^,^48^,^49^,^50^,^51^,^52^,^53^,^54^. Fibrin, alginate, and additional combinations (fibrin/alginate, fibrin/collagen) were investigated, with fibrin serving as the primary material based on prior successful implementation for ovarian tissue transplantation^38^. The primary endpoint of the encapsulation studies was follicle survival within 2 days after encapsulation, to ensure that the follicles would be viable for future *in vivo* transplantation. The isolation procedure for follicles resulted in an encapsulation of approximately 100 follicles per bead for gels with both high and low concentrations of fibrin (Fig. 1a). After two days of culture, the survival of these encapsulated follicles ranged from 53% to 71% (Fig. 1a), with 20 mg/mL having a significantly greater follicle number (p = 0.04). The density of primordial follicles declined over 2 days of culture in both high-density (Fig. 1b) and low-density (Fig. 1c) fibrin, with a greater decline in the high-density fibrin. Primary and secondary follicle densities did not significantly change during the two-day culture for either density of fibrin tested. Alginate was employed as a reference for fibrin, as alginate has been widely used for the culture of secondary and pre-antral follicles^42^, and to a lesser extent, primary follicles^55^. The encapsulation of follicles within the alginate beads was lower than with fibrin (about 60 follicles per bead), likely owing to the higher viscosity of the gel contributing to follicle loss during transfers. In alginate, survival after two days of culture was high, similar to fibrin hydrogels (Fig. 1d). The density of primordial follicles declined during the two-day culture in the 2% alginate (p = 0.01) (Fig. 1e) and 1% alginate (Fig. 1f). An increase in primary follicle numbers was observed in 2% alginate (p = 0.01) (Fig. 1e), but not 1% alginate (Fig. 1f).

**Figure 2:**
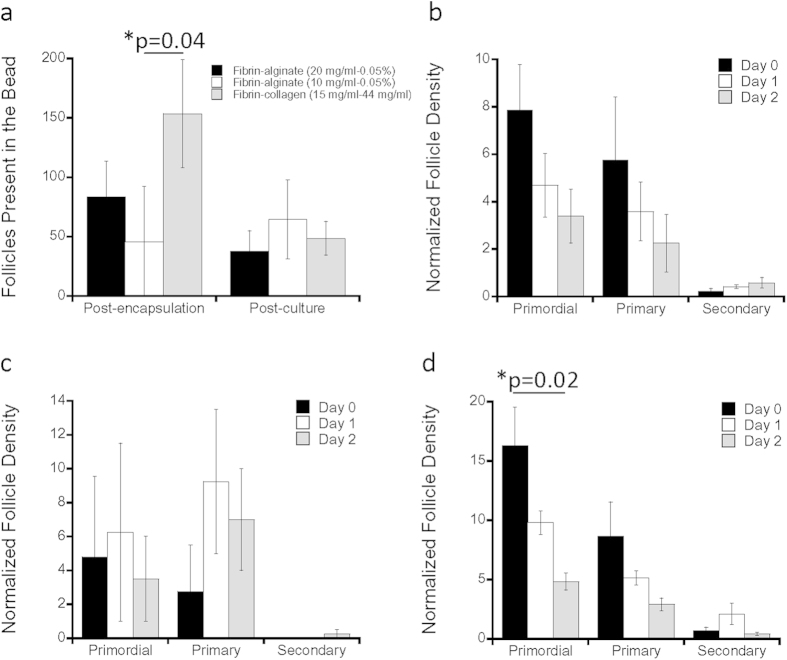
*In Vitro* Follicle Culture in Combination Biomaterials. (**a**) Average absolute number of follicles present in the bead are quantified for post-encapsulation and post-culture in fibrin-alginate (20 mg/ml–0.05%), fibrin-alginate (20 mg/ml–0.05%), and fibrin-collagen (15 mg/ml–0.44 mg/ml), where error bars represent propagated error, with p-value determined by two-tailed Student’s t-test and n is as indicated in (**b**,**c**) and (**d**); follicle quantification in terms of their maturation stage (primordial, primary or secondary) vs. average normalized follicle density with (**b**) fibrin-alginate (20 mg/ml–0.05%) (n = 4); (**c**) fibrin-alginate (10 mg/ml–0.05%) (n = 4); (**d**) fibrin-collagen (15 mg/ml–0.44 mg/ml) (n = 4) over days 0, 1, 2, where error bars represent s.e.m. with p-value determined by two-tailed Student’s t-test; * indicate p ≤ 0.05, for (**d**) comparison is between day 0 and day 2.

Subsequent studies investigated follicle encapsulation and survival in hydrogels based on fibrin combined with a second component, which was incorporated as a means to slow degradation in order to better support the follicles post transplantation^38^. Combinations of fibrin and alginate (FA), as well as fibrin and collagen (FC) had encapsulation and survival rates comparable to fibrin (Fig. 2). The FA conditions tested include 20 mg/mL fibrin with 0.05% alginate and 10 mg/mL fibrin with 0.05% alginate, and are denoted as FA (20/0.05) or FA (10/0.05) respectively. The encapsulation of follicles into FA combinations ranged from approximately 80 to 40 follicles per bead for FA (20/0.05) and FA (10/0.05) respectively, whereas the encapsulation was significantly increased with FC (Fig. 2a, p = 0.04). The survival after two days of culture was 45% for FA (20/0.05), nearly 100% for FA (10/0.05), and 32% for FC (Fig. 2a). The densities of primordial (p = 0.02), and primary follicles declined for FC with FA (20/0.05) or FA (10/0.05) not having a significant change (p > 0.05).

### *In Vivo* Follicle Transplantation within Biomaterials

**Figure 3:**
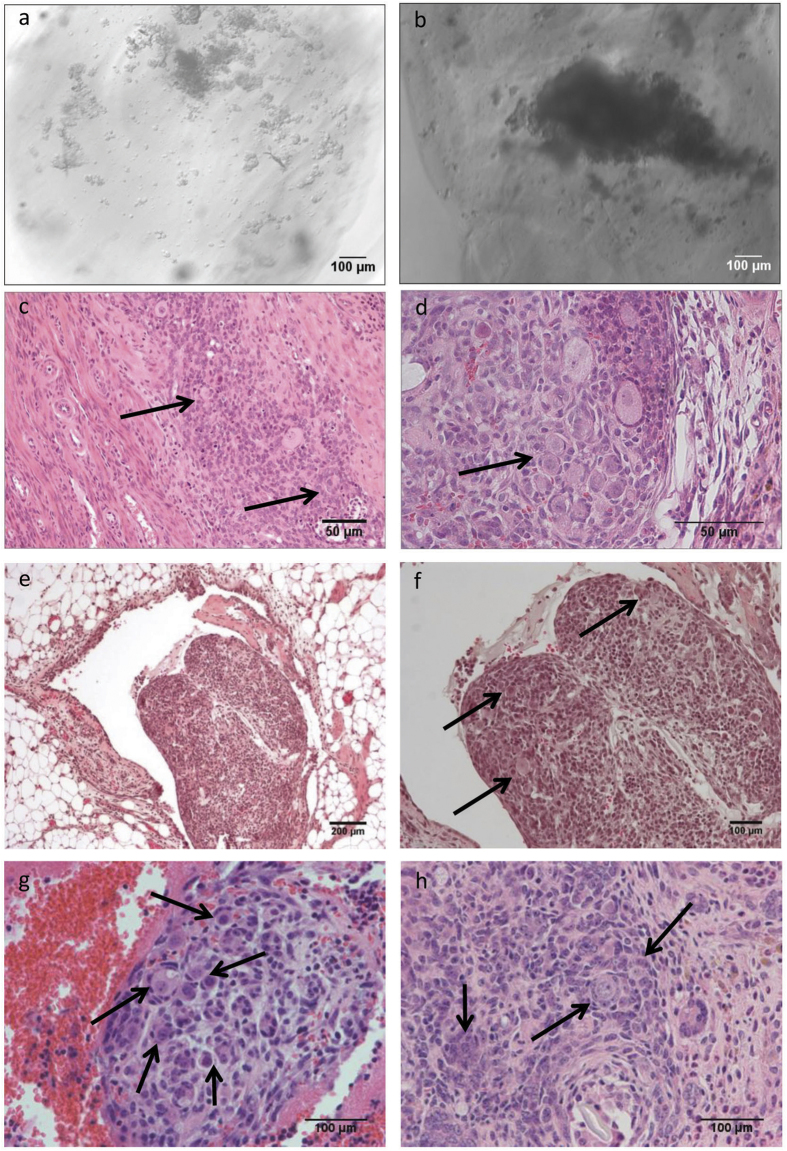
Follicles Survive Transplantation: follicles are present in beads that were prepared by using extra sedimentation procedure to aid in follicle aggregation. The contrast is seen with much sparser follicle population present where follicle-to-follicle contact is minimal in pre-(**a**) and post-(**c**) transplantation (Day 3) beads in fibrin-alginate, as opposed to sedimented tissue pre-(**b**) and post-(**d**) transplantation beads; an example of modified procedure for transplant fabrication where follicles are readily visible within fibrin-alginate transplant surrounded by fatty tissue (**e,f**), within fibrin (**g**) and fibrin-collagen (**h**).

Three material conditions were selected for investigating their ability to support *in vivo* follicle transplantation. Fibrin (20 mg/ml), FC, and FA 10/0.05 were selected as all supported follicle encapsulation, and the inclusion of collagen or alginate was expected to slow the degradation rate of the material relative to fibrin alone. The three biomaterial conditions were initially transplanted within the broad ligament, a relatively accessible site, in order to evaluate the feasibility of transplantation, as well as the integrity of the biomaterial beads. After isolation, follicles were suspended within the hydrogel materials with gentle mixing to distribute the follicles throughout the material (Fig. 3a). Histological staining of retrieved implants revealed that few follicles were present within the graft (Fig. 3c). Consequently, the encapsulation procedure was modified with a sedimentation step, which resulted in an accumulation of follicles near the bead periphery (Fig. 3b), proximal to the host tissue. After inclusion of the sedimentation step, retrieved grafts contained a much larger population of follicles (Fig. 3d).

**Figure 4:**
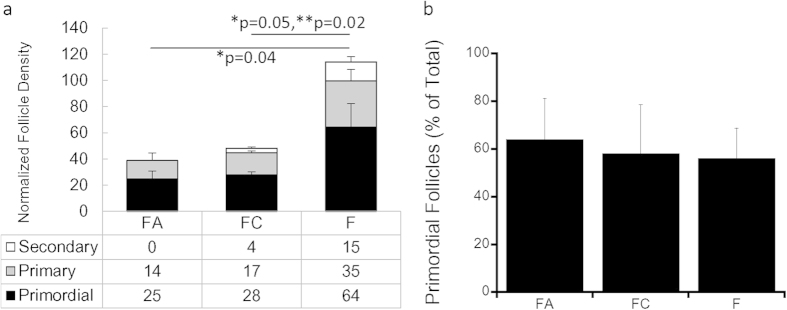
Follicle Population Data for *In Vivo* Studies. (**a**) Average number of follicles (normalized by number of ovaries used per transplant) 9 days post-transplantation represented by secondary, primary and primordial stages for fibrin-alginate (10 mg/ml–0.05%) (FA) (n = 4), fibrin-collagen (15 mg/ml–0.44 mg/ml) (FC) (n = 6), and fibrin (20 mg/ml) (**F**) (n = 7) conditions; error bars indicate s.e.m. with p-value determined by two-tailed Student’s t-test as indicated; (**b**) % primordial follicles in FA, FC and F conditions 9 days post-transplantation, error bars indicate propagated error; * indicates p ≤ 0.05 for comparison of primary follicles, and ** for secondary follicles.

The ovarian bursa was employed for all subsequent studies, with a large number of follicles observed by histology in the grafts (Fig. 3e–h). Quantification of the follicle populations revealed follicle survival and growth had occurred within the fibrin hydrogel (Fig. 4a). At day 9, which was previously reported as supporting a pool of functional follicles^38^, the FA 10/0.05 and FC conditions had a normalized follicle number of 39 and 49 follicles respectively. Within these two conditions, the majority of follicles were primordial, with greater than 50% of primordial follicles, 35% primary follicles, and less than 10% were secondary follicles (Fig. 4b). For the fibrin condition, the normalized follicle number was more than 2–fold greater than either FA or FC (p ≤ 0.05). The majority of follicles within the graft were primordial (56%), with approximately 30% of the follicles in the primary stage, and 13% were secondary follicles (Fig. 4b).

### Mating Studies

**Figure 5:**
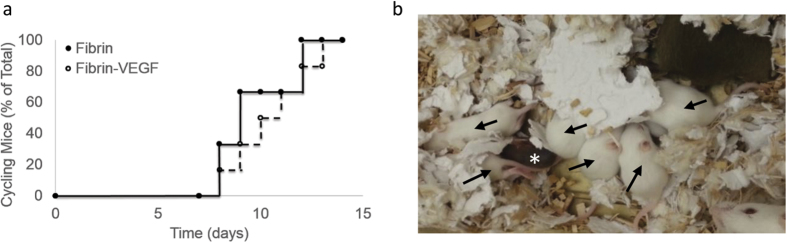
Follicle Transplant Results in a Live Birth. (**a**) Vaginal Cytology Demonstrates Cyclicity in Transplantation Mice: % cycling mice is shown as a function of time over the course of 2 weeks for both fibrin and fibrin-VEGF conditions, where n = 12. 0 time point indicates the onset of cytology measurements, which occurred 4 days post-transplant surgery, when the mice were fully recovered; (**b**) photograph of the mixed litter with both white and grey pups present, arrows indicate white pups, * indicates grey pup.

Mice receiving follicle transplants were subsequently mated to determine the potential for obtaining a live birth. Follicles were transplanted within fibrin gels, and the use of vascular endothelial growth factor (VEGF) was investigated, as VEGF had previously been found to promote live births in ovarian tissue transplantation^38^. The follicle donor and transplant recipient were albino C57/Bl6 mice and C57/Bl6 mice respectively, which were employed to avoid rejection of transplanted tissue, yet allow pups from donor follicles to be identified based on the presence of a white coat. Six females were tested for each of the two biomaterial conditions. Post-transplantation, all females exhibited cyclicity, demonstrated by vaginal cytology (Fig. 5a), with cyclicity occurring as early as day 12 and all mice cycling by day 17. For the 6 female mice transplanted with the fibrin/VEGF condition, three litters were produced, with the first two litters coming from one transplant recipient. Each of the first two litters consisted of a single white pup, with the coat color indicating the transplanted tissue was responsible for the birth. The third litter, from a second transplant recipient, had 6 white pups (from transplanted tissue, 3 males and 3 females) and one grey pup (from residual host tissue) (Fig. 5b). From this litter, the grey pup and one of the white female pups died on day one, yet the remainder of the offspring were healthy and thriving, with no obvious signs of disease at up to 8 months of age. After continuously mating 2 of the remaining females, 4 and 7 healthy litters were produced respectively to date. For follicle transplantation in the fibrin alone condition, no grey or white pup live births were observed. Overall, the minimum number of days from the initial pairing until the birth of a litter was 66 days, the maximum was 143 days, and the average was 102 days, with 2 out of 6 or 33% of females delivering an offspring.

### Removal of Cancer Cells from Transplant

**Figure 6:**
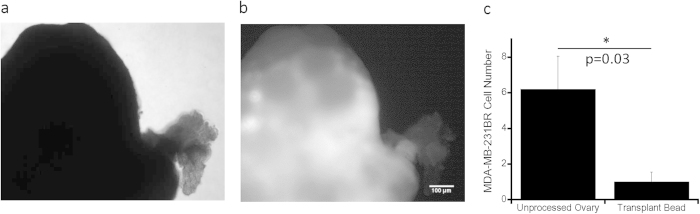
Tumor Burden Evaluation within Unprocessed Ovary and Prepared Transplantation Bead. Cancerous ovarian tissue extracted from a mouse 28 days post-inoculation (phase contrast in (**a**)) is showing a strong red fluorescent signal (**b**); (**c**) flow cytometry results for TdTomato + MD-MBA-231BR cells within unprocessed ovary and transplant bead made from processed ovary, where tissue was extracted 5 days post-inoculation; data shown represent average cell number ± s.e.m., n = 5 and p = 0.03 by two-tailed Student’s t-test, * indicates statistical significance, where p ≤ 0.05.

To evaluate the ability of our procedures to reduce the presence of cancer cells in the graft, NOD-SCID gamma mice with breast cancer xenografts were used as ovarian tissue donors. Ovaries were initially extracted at day 28 after inoculation of cancer cells and imaged to visualize cancer cells, which were identified due to the presence of a tdTomato transgene in the MDA-MB-231BR cells. The ovaries from mice at 28 days post tumor cell inoculation contained numerous 231BR cells as seen via fluorescence imaging (Fig. 6a,b), indicative of a later stage of disease where fertility preservation is not commonly recommended. For an ovary removed at day 5 post-inoculation of tumor cells, the fluorescence signal was insufficient to identify the presence of cancer cells (not shown). The inability to detect the fluorescence signal by imaging suggested low burden of disease, which was further investigated by flow cytometry applied to the ovary, resulting in approximately 6 tumor cells per ovary (Fig. 6c). Ovaries from mice day 5 post-inoculation of tumor cells were subsequently processed for the isolation of follicles and encapsulation within fibrin. Two of the 5 tested beads did not have cancer cells, and the average was 1 cancer cell per implant, a significant reduction relative to the total numbers of tumor cells in the ovary (Fig. 6c).

## Discussion

In this report, we examined a strategy for transplantation of ovarian follicles within biomaterials, which represents a step towards development of a novel means of fertility preservation for young cancer patients. The initial components of a functional transplant included successful encapsulation of follicles within a biomaterial graft. Ovaries were first isolated from 5 to 8-day old C57Bl/6J mice and subjected to 3 rounds of alternating chemical and mechanical digestion. The presence of follicles was verified with phase microscopy and the resulting solution of follicles was encapsulated into a biomaterial. We examined materials based on fibrin as a central component, and mixed fibrin with non-degradable alginate, or more slowly degradable collagen. *In vitro* studies demonstrated that the follicles survived isolation and encapsulation. Relative to the transplantation of ovarian tissue, the transplantation of early-stage follicles is challenged by the potential for disruption of cell-cell interactions during isolation and encapsulation. The biomaterial support can function to maintain the cell-cell interactions following transplantation and thereby enhance follicle survival and promote growth.

In addition to supporting the follicle, the biomaterial functions as a conduit between the host and the graft, in which host cells adjacent to the graft can infiltrate the material and surround and maintain the developing follicles, thereby supporting early and sustained follicle development. We and others have reported that fibrin supports the transplantation of modest numbers of follicles^44^,^56^,^57^,^58^. We also investigated the hypothesis that modification of fibrin with other materials that further slow the rate of degradation would more effectively support early-stage follicles relative to fibrin alone. Fibrin modified with alginate, which has been used for follicle culture, supported follicle survival following transplantation, though minimal cell infiltration was observed in the hydrogel at day 9. Incorporation of collagen, which has also supported follicle growth *in vitro*^59^, into fibrin hydrogels had survival that was comparable to FA, yet was substantially less sustained than fibrin alone. The large number of surviving follicles observed within fibrin hydrogels was the basis for further study of fibrin hydrogels in subsequent breeding studies.

Transplantation of ovarian follicles within fibrin hydrogels demonstrated functionality of the graft, as evidenced by the resumption of cycling and live births. Transplants were performed with and without VEGF, as prior reports using VEGF showed enhanced vascularization within transplanted ovarian tissue in mice. A previous report with the dissociation of the ovary to single cells, with re-aggregation into a fibrin clot for subsequent transplantation, produced live births^56^. Half of the recipients demonstrated estrogenic activity, and approximately 20% produced live births. For the studies herein, after follicle transplantation, all mice resumed cycling in under 3 weeks. Our findings are consistent with prior work showing that follicles surviving transplantation became surrounded by host stromal tissue, and were able to develop to secondary and antral stages *in vivo*, and produced corpora lutea following ovulation^44^. In our study, three separate litters resulted from the transplanted follicles, showing the potential of our biomaterial constructs to provide an environment that supports full follicle function. Moreover, the fact that a single mouse was able to product two consecutive litters showed that the transplanted follicles are setting up physiologic signaling to permit normal follicle selection and potentially avoid universal activation, which is a purported occurrence in ovarian tissue transplants^60^,^61^.

The live births reported herein likely resulted from the superior numbers of engrafted follicles and the delivery of VEGF, which was not investigated in a previous report with the transplantation of ovarian follicles^44^. VEGF delivery may contribute to survival and engraftment of the transplanted follicles, which may be particularly relevant within the context of chemotherapy induced injury that can cause ovarian cortical fibrosis and damage blood vessels^62^. Additionally, we note that live births were achieved in 2 of 6 transplanted mice, despite the observation that all mice resumed cycling. The lack of live births in 4 of the 6 mice may reflect technical issues with the surgical procedure, which may require specific placement of the follicles in order to enable ovulation and transport of the oocyte into the fallopian tubes. Furthermore, poor follicle quality in the graft may underlie graft failure. Although a large number of follicles was transplanted, the quality may have been affected by the isolation and encapsulation process, and/or the post-transplantation environment.

Additionally, ovarian tissue transplantation carries a risk of re-seeding disease^58^,^63^,^64^ and we investigated the extent to which our follicle isolation and encapsulation procedures could remove cancer cells from ovarian tissue. Mice with metastatic breast cancer were used as ovarian tissue donors, where the cancer cells were labeled with a red fluorescent tag, which enabled tracking of the cell number and distribution at various stages of transplantation. A combination of chemical and mechanical digestion procedures were employed to separate stromal cells from early stage follicles, which contrasts with the complete ovary disaggregation and re-aggregation that was used in the previous report of a live birth^56^. The process used herein substantially reduced, but did not eliminate, stromal cells from the follicle. The density of cancer cells in the graft was reduced more than 7-fold relative to the density of cancer cells in the ovary. Two grafts had no residual cancer cells, with the remaining grafts having between 1 and 3 cells. While this procedure did significantly reduce the risk of re-transplanting cancer cells, further development of the isolation and encapsulation procedure is needed to completely eliminate cancer cells. A potential approach could be the introduction of an additional step in which the digested tissue is incubated within a protein or antigen-treated plate that would bind a specific type of cancer cell, consequently only unattached follicles would be removed. Previous work by Schroder *et al.* demonstrated the possibility of tumor cell purging in the setting of a suspension of ovarian tissue, where epithelial tumor cell lysis was achieved by cytotoxic T cell retargeting through the bispecific antibody BIS-1, with combined affinity for the T-cell receptor and epithelial glycoprotein-2 (EGP-2)^65^. Alternatively, screens or sieves could be employed in which the digested ovarian tissue is filtered, and single cancer cells would pass while follicles would be retained. The distribution of cancer cells within the ovary could also be a consideration in the isolation process. If these cells were primarily localized within the vasculature, perfusion of the ovarian prior to follicle isolation and encapsulation may enable a further reduction in the presence of cancer cells. Finally, a recent study showed that simple rinsing of the human follicles was able to remove malignant cells^66^.

Cryopreservation of ovarian tissue and subsequent autotransplantation is an emerging approach for preserving fertility and maintaining endocrine support for patients who are at risk of losing ovarian function, either due to genetic factors, exposure to gonadotoxic radiation or chemotherapies, or other non-malignant diseases and treatments that affect the reproductive axis. Ovarian tissue transplantation has preserved fertility with restored endocrine function; however, for cancer patients, transplantation of ovarian tissue carries a risk of re-seeding disease. To address this limitation, the transplantation of isolated ovarian follicles was investigated, with biomaterials being employed to support the engraftment and function of the transplanted follicles, and resulted in live births. While the current approach reduces the risk of re-seeding cancer cells, additional developments are needed to eliminate this risk and ultimately fulfill the potential for restoring fertility and endocrine function through the translation of follicle autotransplantation for patients surviving cancer.

## Materials and Methods

### Ovarian Digestion

Ovaries were dissected from 6-day old C57BL/6j x CBA/Ca female mice (Harlan Laboratories, USA) to ensure predominantly primordial follicle population, with prior reports indicating that the percentage of primordial, primary, and secondary follicles in the ovaries of C57/Bl6 mice at this age is 80%, 20%, and <0.2% respectively^43^. Upon isolation, follicles were contained in Leibovitz’s L-15 medium (Gibco, USA) for pH control at ambient levels of CO_2_, on a 37 °C heated stage, and on a sterilized bench to minimize bacterial contamination. Ovarian digestion was then carried out under the same conditions in 9-well glass plates in three consecutive steps. For the creation of one graft for transplantation, the following steps were taken: six ovaries were placed in a first glass well containing 500 μL of L-15 media mixed with 1% liberase (Roche Applied Science, Germany) and incubated for 13 minutes. Next the ovaries were rinsed in 500 μL of L-15 in a second glass well for approximately 5 seconds. The ovaries were then transferred to the third well containing 500 μL of L-15 with 5% fetal bovine serum (FBS, Gibco) where they underwent mechanical digestion through repeated pipetting for 4 minutes. This procedure was repeated twice, with the second chemical digestion time reduced to 7 minutes, and the third to 4 minutes. Finally, FBS was added to a final concentration of 10% after the 3^rd^ repeat.

### Incorporation of Follicles into Biomaterials

The incorporation procedures were derived from previously established protocols^36^,^37^,^38^,^50^. Utilizing the dissecting microscope, the digested mixture was transferred to a microtube, where the follicles underwent a series of sedimentation steps. Follicles were allowed to sediment for 15 minutes, after which all but 200 μL of the mixture was aspirated. The follicles then underwent sedimentation for an additional 15 minutes prior to removing 195 μL of the mixture. Next, the biomaterial mixture (5 μL) was combined with the 5 μL of the follicle mixture. After mixing, the follicles underwent a final sedimentation step.

The biomaterial mixtures were prepared as follows: for the fibrin only condition, 20 mg/mL fibrinogen solution was created by combining a stock solution of 40 mg/mL of bovine fibrinogen (Calbiochem, Germany) with phosphate-buffered saline (PBS, Gibco). For the fibrin/alginate condition, the stock solution of 40 mg/mL of fibrinogen was combined with a stock solution of 0.2% alginate (NovaMatrix, USA) at a 1:1 ratio. Adding the materials to the follicle mixture at a 1:1 ratio resulted in a final concentration of 10 mg/mL of fibrinogen and 0.05% alginate. The fibrin/collagen condition was made by combining a stock solution of fibrinogen with 0.44 mg/mL of rat-tail collagen (BD Biosciences, USA). Several concentrations of these biomaterials were tested. Finally, 10 μL of the combined follicle-biomaterial solution was mixed with 50 μL of thrombin/Ca^2+^ (Sigma-Aldrich, USA) solution to initiate cross-linking. The thrombin/Ca^2+^ solution was prepared by combining 50 IU/mL thrombin with 40 mM CaCl_2_. After 5 minutes, the beads were transferred from the microtube and stored in a petri dish containing warmed L-15 media. Prior to transplantation or *in vitro* culture, beads were imaged to confirm the presence of follicles, evaluate follicle size and also maturation stage.

### Ovariectomy and Transplantation

Beads were transplanted into adult isogenic female mice (C57BL/6j x CBA/Ca). Mice were first anesthetized via intraperitoneal injection of mixture of a 100 mg/kg of ketamine and 15 mg/kg of xylazine. The uterine horn, oviduct, and ovary were localized, and beads were transplanted either into the broad ligament or into the ovarian bursa. For the broad ligament procedure, the beads were carefully placed within a small pocket created within the ligament, and then sutured (10-0 Nylon, Angiotech, British Columbia) in place. For the bursa transplants, the ovarian branches of the ovarian artery and vein were ligated using 10-0 Nylon suture. An incision was then made along the ovarian bursa, which was carefully reflected to expose the underlying ovary, and the ovary was then excised. A bead was then placed within the cavity and the bursa closed using 10-0 Nylon suture. This process was repeated on the contralateral side and the abdomen closed in two layers using 5-0 Monocryl suture (Angiotech). Mice were also administered 2 mg/kg of Buprenorphine subcutaneously in the post-surgical period for analgesia.

### Retrieval of Transplanted Beads and Histological Processing

After 9 days, the mice were euthanized and the ovarian bursae containing the transplanted beads were retrieved for histological staining. The tissue was fixed in 4% paraformaldehyde, paraffin-embedded, and serial-sectioned prior to undergoing hematoxylin and eosin staining. The total number of follicles per transplant and quality of follicle populations were analyzed by two independent researchers who were blinded to the treatment conditions. The normalized follicle number was reported for these studies, which referred to the total number of counted follicles divided by the number of ovaries used to construct an individual biomaterial bead.

### Breeding Studies and Live Birth

The potential for the transplanted follicles to support a live birth was investigated. C57Bl/6 albino mice (C58BL/6/BrdCrHsd-Tyrc) (Harlan Laboratories) served as the follicle donors, and transplant recipients were C57Bl/6xCBA F1 hybrids. The recipients were mated with CD-1 outcross albino males (Harlan Laboratories). 6 females were used for each biomaterial condition, for a total of 12 females tested within fibrin and fibrin-VEGF. Preliminary studies indicated that the ovarian tissue would not be rejected in this model, and that the offspring would have a white coat distinct from the offspring of C56Bl/6xCBA F1 hybrid crossed with CD-1 mice. After 4 days once the mice were fully recovered post-transplantation, vaginal cytology was performed for two weeks to verify recipients’ cyclicity. Once the cycling was established, the mice were then continuously paired with CD-1 male breeders for the course of at least 6 months. All offspring remained with the mother until weaning.

### Cancer Model

The follicle donors were obtained by inoculating human MDA-MB-231-BR (231-BR) cells, a highly metastatic estrogen and progesterone receptor and HER2/neu receptor negative (triple negative) breast cancer cell line^67^, into the 4th mammary fat pad (MFP) of NOD-SCID gamma (NSG) mice up to 6 weeks of age. The 231-BR cells were engineered to express tdTomato, a red fluorescent protein. Ovaries were removed at 5 days post-inoculation and biomaterial beads constructed as previously described, with one ovary used to produce each bead. Flow cytometry analysis was performed on 5 ovaries from distinct mice retrieved at day 5, as well as for 5 biomaterial beads created from corresponding retrieved ovaries. Data was reported as average cell number ± s.e.m., with p-value determined by paired Student’s t-test. Fluorescence and phase contrast images were obtained from intact ovaries 28 days post-inoculation to demonstrate the extent of cancer cell burden at a later stage of cancer progression when the fluorescence signal was clearly visible.

### Study Design and Conditions

*In vitro* culture studies were performed on 8 material concentrations, with a total of 4 repeats for each condition. Each biomaterial bead required 6 donor ovaries acquired from C57BL/6j x CBA/Ca female mice. *In vivo* studies where biomaterial conditions were tested, were performed for 3 material conditions, 1 time point each, with a total of 4 repeats, and also required 6 donor ovaries to construct each transplant (each recipient received 2 biomaterial beads). Cancer cell studies were performed with 5 animals, each requiring one donor mouse. Finally, live birth studies were performed with 12 animals, with similar conditions as described above.

### Statistical Analyses

Both *in vitro* and *in vivo* data were collected for n ≥ 4, as indicated. The error bars represent standard error of the mean (s.e.m.) for Figs 1,2,4 and 6, except 1a, 1d, 2a, and 4b, where propagated error was reported. Histological evaluation was performed by two independent researchers who were blinded to the experimental condition, with the consensus of data outcomes verified. For statistical analysis, multiple comparisons were performed with accounting for repeated measures using one-way ANOVA followed by a Student’s t-test, or a t-test alone for cases with only two condition comparisons, with significance defined as a p-value ≤ 0.05.

### Ethical Approval

Animal research carried out in this manuscript was performed under the written approval of the Northwestern University Institutional Animal Care and Use Committee (IACUC) in accordance with all federal, state, and local guidelines. Specifically, experiments on animals were performed in accordance with the guidelines and regulations set forth by the National Institutes of Health Guide for the Care and Use of Laboratory Animals and the established Institutional Animal Use and Care protocol at Northwestern University.

## Additional Information

**How to cite this article**: Kniazeva, E. *et al.* Primordial Follicle Transplantation within Designer Biomaterial Grafts Produce Live Births in a Mouse Infertility Model. *Sci. Rep.* **5**, 17709; doi: 10.1038/srep17709 (2015).
